# Evaluation of a University-Based Certificate Program Designed to Bolster the Workforce of Implementation Support Practitioners

**DOI:** 10.1177/26334895261456681

**Published:** 2026-06-04

**Authors:** Todd M. Jensen, Allison J. Metz, Mackensie E. Disbennett, Jenny L. Afkinich, Amanda B. Farley

**Affiliations:** 1 School of Education, 2331 University of North Carolina at Chapel Hill, Chapel Hill, NC, USA; 2 School of Social Work, 279022 University of North Carolina at Chapel Hill, Chapel Hill, NC, USA; 3 Department of Social Work and Sociology, 3616 North Carolina Agricultural and Technical State University, Greensboro, NC, USA

**Keywords:** Certificate program, competencies, implementation, implementation practice, implementation research, implementation science, training

## Abstract

**Background:**

There is an increasing demand for building a workforce capable of using evidence generated from implementation science to support the wide-scale use of evidence-informed interventions to achieve equitable outcomes. This study focuses on the feasibility of growing the competencies of professionals who actively support change efforts in service systems—referred to as implementation support practitioners—through a synchronous, university-based, online certificate program in implementation practice.

**Method:**

The certificate program consisted of three 2-day courses, each grounded in 15 core competencies linked to skillful implementation support and delivered using an online synchronous approach to three cohorts of participants between February 2023 and May 2024. Enrollment for each cohort was limited to ensure personalized interaction between program faculty and participants, with cohort sizes ranging from 36 to 47. Leveraging a mixed-methods approach, we collected and analyzed both quantitative and qualitative evaluation data to evaluate the certificate program.

**Results:**

A large majority of participants from all three cohorts agreed that they were satisfied with program and experienced gains in knowledge, skills, and perceived capability, motivation, and opportunity to apply their learnings. Participants also yielded significant gains from pre- to post-program across all 15 targeted competencies. Qualitative analyses indicated that participants appreciated the program's high-qualty faculty, skilled facilitation, respectful learning environment, and practical curriculum. The synchronous format and consistent small-group work also were valued. Suggested program improvement included more small-group time and clearer links between readings and speaker content. Follow-up surveys indicated sustained impacts and content application in participants’ day-to-day work activities.

**Conclusions:**

Alongside other extant training approaches, university-based, competency-focused certificate programs delivered using an online synchronous approach could offer an effective strategy for bolstering the workforce of implementation support practitioners. Efforts on this front could aid in reducing a growing divide between implementation research and practice.

## Plain Language Summary

An Initial Evaluation of a University-Based Certificate Program Designed to Train More Professionals Who Help Implement Programs, Policies, and Practices in Health and Human Service Settings

There is a dearth of studies on implementation science capacity-building programs for nonresearchers. Specifically, recent reviews point to the lack of capacity-building and training programs for professionals who support service providers to implement evidence-based programs, practice, and policies. This study assesses the acceptability and effectiveness of a university-based certificate program in implementation practice for professionals who provide implementation support. The results are promising, with program participants showcasing notable and sustained gains in implementation-related knowledge, skills, motivation, capability, and opportunity. This study addresses both a gap in the literature and the growing divide between implementation research and practice.

## Introduction

The field of implementation science has rapidly grown over the last two decades, contributing to a better understanding of the factors that lead to successful implementation (Westerlund et al., 2019). With this growth, there is an increasing demand for building a workforce capable of using evidence generated from implementation science to support the wide-scale use of evidence-informed programs and practices to achieve equitable outcomes (Metz et al., 2021). The demand for such a workforce has resulted in two areas of inquiry—the identification and operationalization of competencies for implementation scientists, including researchers and practitioners, and the development of training and capacity-building programs for professionals seeking to develop these competencies. This study addresses both of these areas of inquiry with a specific focus on the feasibility of growing the competencies of professionals who actively support change efforts in service systems—referred to as implementation support practitioners (Albers et al., 2020; Metz et al., 2021)—through a fully synchronous, university-based, online certificate program in implementation practice.

While there is increasing attention for scaling the benefits of implementation science through capacity-building programs, the literature has drawn attention to the limited number of capacity-building programs for non-researchers. A recent study of implementation science capacity-building programs reflected the current state of such programs, which focus almost exclusively on implementation researchers (Huebschmann et al., 2022). These programs train implementation researchers on the use of research methods grounded in specific implementation theories, models, and frameworks. The dearth of capacity-building programs for implementation practitioners is complicated by the lack of a common definition of implementation practitioners; descriptions of implementation practitioners range from clinical practitioners in direct practice to professionals who support implementation and change efforts in health and human service settings.

A recent systematic review of dissemination and implementation science capacity-building programs found that few programs endorsed offering implementation science training within clinical professional degree programs (Viglione et al., 2023), reinforcing the limited capacity-building opportunities in implementation practice. However, this systematic review did not mention professionals who work at the nexus of research and clinical practice—professionals often referred to as implementation support practitioners (Albers et al., 2021; Metz et al., 2021).

Implementation support practitioners support others to implement evidence-informed practices, programs, and policies to achieve population outcomes (Albers et al., 2020). This group of individuals was highlighted in the Interactive Systems Framework (Wandersman et al., 2008) as comprising the “implementation support system” that is needed to connect research and synthesis activities with direct service changes. Implementation support practitioners are not involved in direct service delivery (i.e., clinical practitioners) or management, but work closely with leadership, staff, and implementation teams to deliver educational, clinical, and therapeutic services to populations and communities. Implementation support practitioners can reside *outside or inside* the system they are supporting (Metz et al., 2021). Wandersman et al., (2008) describe how implementation support systems can facilitate these goals. Participants can leverage the competencies they gain through the certificate to partner with colleagues in these internal units to serve in these implementation support roles. In order to provide this support effectively, implementation support practitioners rely on their knowledge and ability to identify, tailor, and improve evidence-based implementation strategies to ensure high quality, consistent service changes and to use implementation science theories, models, and frameworks to appropriately diagnose implementation barriers and develop plans for addressing these barriers.

Recent research on training programs for implementation support practitioners has found promising results. For example, Kirchner et al., (2022) described the development and preliminary results of a training program for implementation facilitators who engage in similar activities as implementation support practitioners. Findings from this study demonstrate that a 2-day training program (in-person or virtual) can increase participants knowledge and confidence in 12 implementation facilitation competencies. An earlier study (Moore et al., 2018) of a training program for implementation support practitioners also demonstrated increases in self-reported knowledge and use of implementation frameworks (Moore et al., 2018). Although these findings are promising there remains a limited focus on professionals who provide implementation support in capacity-building programs, training, and education curricula, leaving a gap in our ability to leverage the benefits of implementation science for wide-scale improvements in service delivery. Furthermore, the operationalization and validation of implementation support competencies have evolved since these earlier studies, providing an opportunity to assess changes in research-based competencies using a psychometrically validated assessment (Jensen et al., 2024).

**Figure 1.**
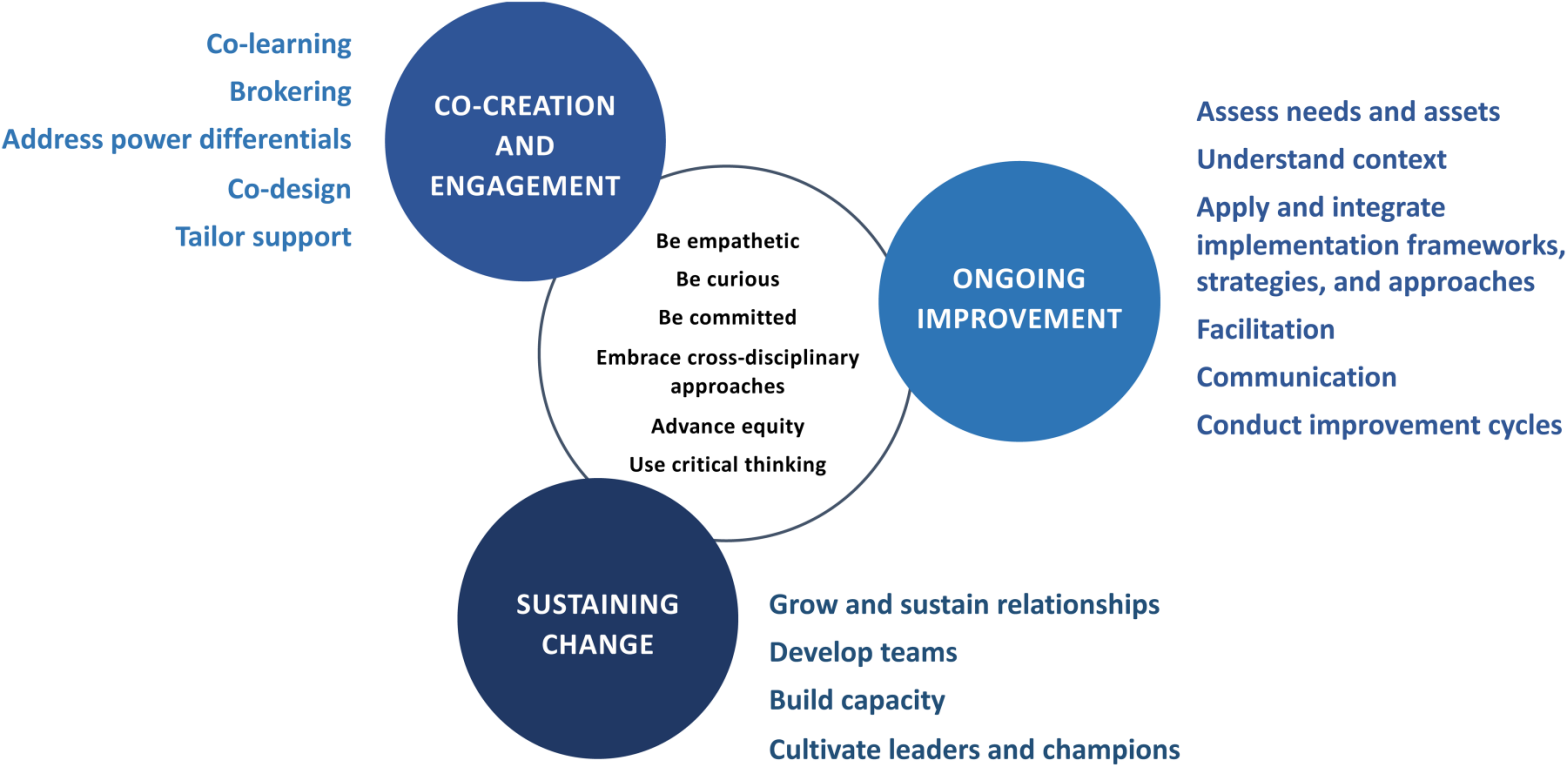
Visual Overview of Evidence-Informed Implementation Support Practitioner Guiding Principles and Core Competencies.

This study focused specifically on a population of implementation support practitioners who engaged in a university-based implementation practice certificate program rooted in a set of competencies identified and empirically studied for this role (see Figure 1 for a visual overview of the competencies) (Albers et al., 2020, 2021, 2022; Bührmann et al., 2022; Metz et al., 2021). An evaluation of this competency-based training program for implementation support practitioners enables further study and substantiation of the competencies defined by Metz and colleagues and can deepen our understanding of optimal processes related to designing and delivering training and capacity-building programs that are focused on specific and distinct professional roles in the field of implementation science—in this case, professionals who use findings from implementation research to improve implementation practice. This training program also provides an opportunity to (a) professionalize a subset of the implementation science workforce that focuses on implementation practice and (b) build community among these professionals where ongoing learning can take place to improve implementation support strategies. The following research questions governed our evaluation: (1) To what extent were participants satisfied with the program (i.e., acceptability)? (2) To what extent did participants acquire relevant knowledge? (3) To what extent did participants experience gains in their capability, motivation, and opportunity to apply learnings? (4) To what extent did participants acquire gains in key implementation support competencies?

## Methods

### Certificate Program Description

#### Content and Format

The certificate program was designed for leaders, managers, and staff working in health and human services who support change efforts, including delivery and implementation of programs, practices, and policies to improve outcomes and advance equity. Curriculum content was firmly grounded in a set of 15 core competencies (organized by three over-arching domains) linked to skillful implementation support. As noted earlier, the competencies were developed on the basis of extensive conceptual and empirical work (Albers et al., 2020, 2021, 2022; Bührmann et al., 2022; Metz et al., 2021). The certificate program in implementation practice is a professional certificate, which does not require the completion of credit-bearing courses and can offer flexibility in delivery models to accommodate busy professionals, while still providing participants with a university-based certificate from a top-five School of Social Work in the United States, continuing education credits, and a digital badge. The certificate program is not a profit-driven model and tuition is set to cover the costs associated with delivering the program so that the program is self-sustaining.

The program consisted of three two-day courses. Course one corresponded with the *co-creation and engagement* domain; course two corresponded with the *ongoing improvement* domain; and course three corresponded with the *sustaining change* domain. The certificate program emphasizes competencies and values for implementation support, rather than endorsing specific frameworks or models, in order to build the capacity of participants to integrate methods and strategies relevant and helpful to the organizations and systems where they support change and evidence-use. For example, content on methods for selecting and tailoring strategies is coupled with skill-building activities such as conducting empathy maps and supporting co-learning among implementation partners, so that the processes for selecting and tailoring strategies integrate the perspectives of a range of community and agency partners. The overarching goal of the program is to explicitly tether implementation science knowledge with implementation practice skills.

Each 2-day course amounted to 9 h of engagement, totaling 27 h across all three courses. The courses were delivered using an online synchronous approach via the Zoom web-conferencing platform. In terms of format, courses incorporated a mixture of didactic content delivery, small-group reflection and application activities, and large group discussion. Two-day courses were delivered on consecutive days. The three courses were delivered over 3 months with an average of 2–3 weeks between courses for participants to complete evaluations and homework assignments and read assigned materials for the upcoming course. Each course is $600 for a total of $1,800 for the full certificate program. Scholarships are provided on a rolling basis with philanthropic support and typically one-third of program participants receive financial support. Tuition covers the delivery cost of a fully synchronous program with intensive facilitation of small groups. The program is self-sustaining and does not make a profit.

The rhythm of each day for each course consisted of reflections from the previous day (after the first day) or previous course, anticipatory guidance for the current day's agenda, at least three large group sessions that involved content delivery, and at least two small group sessions to practice using skills or testing implementation resources through the use of case examples. Each day ended with key take-aways from participants in terms of what learning resonated the most for their implementation practice. In addition to large and small group sessions, the course utilized videos, polling techniques, annotation exercises, and role plays to facilitate interaction among participants and between participants and faculty.

Small groups had consistent membership throughout the program and were facilitated by one of five core program faculty (about nine participants per facilitator). Participant guides were provided for each course, which contained content from course slides, spaces for note-taking, reflection and application activities, and additional resources. In addition to content delivered by core program faculty, nine guest speakers with relevant expertise contributed to content delivery across the three courses. All program materials were housed on the Moodle platform. Program faculty were based at an implementation science center at a School of Social Work either as full time faculty and staff or as long-term consultants. All program faculty were engaged in preparation activities to deliver the program including intensive reviews of small group activities.

The certificate program embraces a pedagogical model that included six phases of faculty engagement and participant learning that take place cyclically throughout each course as each course opens with opportunities for connection and relationship-building in small groups; as content is presented by faculty; as participants are engaged in follow-up small groups to discuss, practice, and reflect on content presented; and as evaluations are completed at course completion. The six phases are explicated in Table 1.

**Table 1.** Six Phases of Faculty Engagement and Participant Learning.

Phase	Description
Connection	Faculty sought to build personal connections with participants by modeling active listening, empathy, checking for understanding, and encouraging questions and new ideas. Connections were fostered through the consistent memberships in small groups and through set agendas and formats that helped participants know what to expect and feel comfortable participating in a range of ways (e.g., chat functions, verbally, polling, annotation, small groups).
Explanation	Participants were exposed to traditional formats of learning where faculty presented on content with the purpose of increasing knowledge and awareness of participants on key implementation science and practice topics.
Observation	Participants had opportunities to observe faculty using specific skills through role plays or applying implementation tools and resources through case examples. For example, faculty demonstrated co-learning skills through the use of sequenced interview questions in the early stages of an implementation support engagement.
Transformation	Participants were invited in small group sessions to both practice newly learned implementation strategies through case examples generated by faculty and participants themselves and to also test out newly learned skills through role plays and behavior rehearsals. These activities helped participants to expand on their understanding of the material. For example, participants had opportunities to practice empathy mapping as a strategy for tailoring implementation strategies for different implementation partners.
Reflection	Faculty and participants reflected together on what happened in large and small group settings and were encouraged to think critically about ways to improve implementation practice. For example, participants were asked to reflect on a recent implementation challenge and what they considered when deciding on how to address this challenge.
Evaluation	Participants were given opportunities to assess their understanding of materials and confidence in providing skill-based implementation support. Participants were required to achieve passing grades on individual knowledge assessments following each course. Participants were also required to respond to qualitative questions asking for reflections on their individual implementation practice and how they may apply concepts and skills from the course in their future work supporting implementation.

Table 2 provides a detailed overview of each course, including the focal domain, associated core competencies, and specific course objectives. Courses for cohort 1 were delivered from early February to late March 2023; courses for cohort 2 were delivered between November 2023 and January 2024; courses for cohort 3 were delivered between March and May 2024. Upon completion of all three courses and related requirements, participants were able to receive a personalized certificate of completion, a digital badge from the University of North Carolina at Chapel Hill, and access to continuing education credits. Additional information about the certificate program is available upon request.

**Table 2.** Certificate Program Overview.

Course domain	Core competencies	Course objectives
Course 1: Cocreation and Engagement	Colearning Brokering Address power differentials Codesign Tailoring support	Name and differentiate between competencies needed to cocreate and engage with a range of implementation partners. Demonstrate an ability to foster co-learning using a one-to-one activity. Give examples of technical and relational strategies for building trusting relationships. Use the spectrum of engagement to identify current levels of engagement for implementation partners and assess whether a different level of engagement is needed. Plan for feasible opportunities to address power differentials and advance equity in their implementation effort. Distinguish how brokering and tailoring support strategies contributed to outcomes in a case example of a change effort. Employ empathy interviews as a method for tailoring support.
Course 2: Ongoing Improvement	Assess needs and assets Understand context Apply and integrate implementation frameworks, strategies, and approaches Facilitation Communication Conduct Improvement Cycles	Name and differentiate between competencies needed to support ongoing improvement of an implementation effort. Explain a process for assessing contextual fit and implementation feasibility of a program, practice or strategy and apply it to a case study. Give examples of implementation determinants and implementation strategies matched to those determinants. Develop a plan that matches implementation strategies to specific implementation challenges. Explain the importance of visually communicating data to guide improvement and decision-making. Examine key activities used to carry out improvement cycles as illustrated by a case example. Choose specific facilitation techniques based on intended meeting goals.
Course 3: Sustaining Change	Grow and sustain relationships Develop teams Build Capacity Cultivate Leaders and Champions	Name and differentiate between competencies needed to sustain an implementation or change effort. Compare and contrast four types of psychological safety for teams including the strategies used to build each type of psychological safety and the various impacts when they’re missing. Recognize the characteristics of effective champions and consider these characteristics when identifying champions. Explore a process for assessing and planning for sustainability of an implementation effort using the Sustainability Planning Tool. Name courageous leadership skills and identify strategies to support use of these leadership skills across implementation teams Describe how values can be used to drive implementation decision-making and examine how values have facilitated or hinder a current implementation effort. Give examples of strategies used to build implementation capacity and relate them to specific outcomes demonstrating whether capacity has been built.

#### Recruitment and Participants

For cohorts 1 and 3, information about the certificate program was advertised via professional listservs and social media only. Advertised cohorts were filled within 2 weeks of announcement, and a waitlist was created for individuals who sought to register after the cohort was filled. Cohort 2 was comprised of a large group of professionals working in the same non-profit setting who expressed interest in completing the certificate program as a large group. Individuals were able to use a web-based university platform to register for the certificate program. Registration included a survey that asked for information about the number of years experience in implementation science for potential participants and the service context within which they work.

The majority of certificate participants worked inside public systems and nonprofits supporting work units specifically designed to support innovation, implementation, improvement and/or scaling efforts. The typical participant is a leader or manager in a public system, nonprofit agency, or community-based organization who leads teams that are responsible for wide-scale change efforts. A few examples of participants include the following: a leader of a public child welfare system who is responsible for supporting teams to use data for quality assurance and quality improvement activities, a technical assistance manager for a federally funded early childhood center that leads teams to implement evidence-based programming in child care settings, and a university-based implementation specialist who leads a center to support evidence-use in healthcare. There is a range of explicit knowledge or experience in implementation science for participants which the program seeks to accommodate. The limited enrollment and small groups allow for tailored approaches to capacity-building. For example, if participants are asked to use an implementation framework to diagnosis implementation challenges in a current project, faculty have the time and space to provide extra guidance to less experienced participants who may need additional support in understanding the framework before beginning the diagnostic exercise. The scaffolding that takes place among more experienced participants with less experienced participants is a key aspect of the program. Enrollment for each cohort was limited to ensure personalized interaction between program faculty and participants. The inaugural cohort (cohort 1) included 44 participants, the second cohort included 36 participants, and the third cohort included 47 participants. Formal completion rates ranged from 86% to 94% across cohorts. Table 3 provides details about participant characteristics for each cohort, including gender identity, racial/ethnic identity, continent of residence, years of professional experiences, service type, and work setting.

**Table 3.** Participant Characteristics.

	Cohort 1		Cohort 2		Cohort 3	
	*N* = 44		*N* = 36		*N* = 47	
Characteristic	*n*	%	*n*	%	*n*	%
Gender identity						
Woman	33	75	35	97	38	81
Man	11	25	1	3	9	19
Racial/ethnic identity^a^						
White	32	73	30	83	34	72
Black/African American/African descent	10	23	4	11	9	19
Hispanic	3	7	2	6	1	2
Asian	2	5	0	0	2	4
Native American/American Indian/Alaska Native	1	2	0	0	1	2
Two or more races	1	2	0	0	0	0
Continent of residence						
North America	37	84	36	100	34	72
Europe	4	9	0	0	8	17
Africa	2	5	0	0	2	4
Asia	1	2	0	0	1	2
Years of experience						
1–5 years	6	14	2	6	9	19
6–10 years	8	18	6	17	5	11
11–15 years	5	11	5	14	9	19
More than 15	25	57	22	61	24	51
Service type^a^						
Public health	8	18	11	31	7	15
Child welfare	14	32	20	56	18	38
Health	6	14	6	17	9	19
Mental and behavioral health	8	18	17	47	14	30
K-12 education	4	9	1	3	6	13
Criminal justice	4	9	2	6	6	13
Implementation science	15	34	14	39	22	47
Early childhood	19	43	24	67	4	9
Other	3	7	3	8	10	21
Work setting^a^						
Faith-based	1	2	0	0	0	0
Federal government	9	20	0	0	5	11
For-profit	5	11	0	0	2	4
Higher education	7	16	0	0	9	19
Hospital	2	5	0	0	3	6
International	3	7	0	0	0	0
Local government	10	23	0	0	6	13
Non-profit	20	45	36	100	15	32
Primary/secondary school	1	2	0	0	2	4
State government	13	30	0	0	13	28
Other	4	9	0	0	4	9

a Percentages can exceed 100% because participants were able to select all responses that applied to them.

### Evaluation

#### Data Collection and Measures

Leveraging a mixed-methods approach, we collected both quantitative and qualitative evaluation data using the following strategies and instruments: (a) web-based evaluation surveys with closed- and open-ended items administered after each course (all three cohorts), (b) the Implementation Support Competencies Assessment (ISCA; all three cohorts), and (c) a follow-up survey (cohorts 1 and 2).

#### Course Evaluation Surveys

Following each of the three courses, participants were issued a course-specific web-based survey with several close-ended and open-ended evaluation items. The survey was administered via Moodle, the course management software used for the certificate program. Consistent with the Kirkpatrick's Model of Learning Evaluation (Kirkpatrick & Kirkpatrick, 2008), a set of close-ended items focused on participants’ (a) reactions to the course (e.g., overall and element-specific satisfaction; perceived quality and usefulness) and (b) self-assessed learning, skill acquisition, and potential impact of course participation. Consistent with the *capability, opportunity, and motivation* behavior change system (COM-B) as articulated by Michie and colleagues (Michie et al., 2011), additional close-ended items asked participants about their perceived capability, motivation, and opportunity to use their learnings in practice. Each close-ended item was presented as a statement to which participants could indicate their level of agreement, with response options ranging from 1 (*strongly disagree*) to 5 (*strongly agree*). In terms of open-ended evaluation items, participants were asked the following: “What did you like best about the course?” “In what ways could the course be improved?” and “What else would you like to share about your experience with this course?”

#### Implementation Support Competencies Assessment (ISCA)

The ISCA is a recently validated, literature-based assessment instrument that is designed to measure the 15 core competencies of implementation support covered in the certificate program (Jensen et al., 2024; Metz et al., 2024). Each competency was measured using a set of items (ranging from 5 to 15) that describe common activities related to that competency (113 items total). Respondents were instructed to reflect on their experiences supporting implementation in various settings, review each item, and assess their level of competence by selecting one of the following response options: (a) not at all competent, (2) slightly competent, (3) moderately competence, (4) very competent, or (5) extremely competent. In the event the respondent did not have direct experience with a particular item, they were encouraged to indicate how competent they would expect themselves to be if they were to conduct the activity described in the item. Prior to each of the three courses, participants were asked to complete ISCA items associated with the domain covered in that course (i.e., pre-program assessment) via Qualtrics, a web-based survey platform. Following the completion of the certificate program, participants were asked to complete the full ISCA once more (i.e., post-program assessment). The post-program ISCA began as a strongly encouraged activity among members of cohort 1, but became a formal requirement (for the receipt of a program completion certificate) for members of cohorts 2 and 3. For purposes of contrasting pre- and post-program responses, we estimated average scores for each multi-item scale at both time-points. Assessments of internal consistency reliability for each multi-item scale yielded evidence of acceptable reliability (i.e., Cronbach's alpha ranged from 0.68 to 0.96 across competencies and cohorts; McDonald's omega ranged from 0.69 to 0.96 across competencies and cohorts; see Jensen et al. (2024) for more details related to the development and psychometric assessment of the ISCA).

*Follow-Up Survey*. In an effort to assess sustained impact and content application following program completion, we issued a brief follow-up survey to participants in cohort 1 (about one year following program completion) and cohort 2 (about three months following program completion). The survey provided concise summaries of each course and asked participants how impactful each course has been for their implementation support work. Response options ranged from 1 (*not at all impactful*) to 5 (*extremely impactful*). The survey also included the following open-ended item: How have you used what you learned in the certificate program in your implementation support work?

#### Analysis

Descriptive statistics were estimated to summarize information about the closed-ended course evaluation survey items. Specifically, as an efficient summary of affirmative responses we estimated the percentage of participants who either agreed or strongly agreed with the evaluation statements provided in the survey. Descriptive statistics were estimated in the form of means and standard deviations to summarize responses to the close-ended follow-up survey items. We also conducted two-tailed, paired-samples t-tests to assess whether average scores for each of the 15 competencies represented in the ISCA changed significantly from pre- to post-program. Preliminary diagnostic analyses were conducted to ensure key data assumptions were not violated (e.g., burdensome distributional skewness or kurtosis). All quantitative data management and analyses were conducted in Stata 17. With respect to the open-ended evaluation survey items and follow-up survey items (i.e., qualitative data), we developed core themes consistent with a qualitative content analysis approach (Schreier, 2014). The focal conceptual categories that guided theme development centered on (a) what participants liked about the program, (b) suggestions participants had for program improvement, and (c) participants’ reports of the sustained value and ongoing use of program content in the context of their work.

#### Ethics Approval

Following our internal review of evaluation data for continuous improvement purposes, we submitted an application (study : 23-1515) to our university's Office of Human Research Ethics, whereby we proposed use of fully de-identified data to share aggregated findings via research publications and related dissemination outlets. Following review, the Office of Human Research Ethics determined that the submitted request did not constitute human subjects research as defined under federal regulations [45 CFR 46.102 (e or l) and 21 CFR 56.102(c)(e)(l)] and did not require Institutional Review Board approval.

## Results

### Quantitative Findings

#### Course Evaluation Surveys

Table 4 provides a summary of responses to closed-ended evaluation survey items. Across all three courses and cohorts, participants demonstrated consistently high agreement rates (agree or strongly agree) with evaluation items related to positive reactions (91%–100%), self-assessed learning and skill acquisition (73%–100%), and COM-B elements including capability, motivation, and opportunity (88%–100%). Examining findings across courses and courses, several patterns were evident. For reaction items (e.g., overall satisfaction, format, content quality, faculty quality and responsiveness, applicability of knowledge), agreement was near-universal (91%–100%) across all courses and cohorts. One unique item assessed following Course 1 pertained to satisfaction with the registration process; 91%–98% of participants across cohorts agreed. For self-assessed learning items, two notable outliers emerged in Cohort 1: 73% agreed they could plan to address power differentials and advance equity (Course 1), and 79% agreed they could develop a plan matching implementation strategies to challenges (Course 2). These same items showed improved agreement in Cohorts 2 (82% and 91%) and 3 (94% and 93%), suggesting successful content refinements. All other learning items achieved near or above 90% agreement across courses and cohorts. For COM-B items assessing perceived capability, motivation, and opportunity to apply learnings, agreement ranged from 88% to 100% across all courses and cohorts, with items related to having adequate supports and resources showing the most variability.

**Table 4.** Summary of Affirmative Responses to Close-Ended Evaluation Survey Items.

	% A/SA					% A/SA					% A/SA		
Course one evaluation items	Cohort 1 (*n* = 41)	Cohort 2 (*n* = 34)	Cohort 3 (*n* = 47)		Course two evaluation items	Cohort 1 (*n* = 42)	Cohort 2 (*n* = 32)	Cohort 3 (*n* = 45)		Course three evaluation items	Cohort 1 (*n* = 40)	Cohort 2 (*n* = 32)	Cohort 3 (*n* = 45)
*Evaluation target: Kirkpatrick model level 1—reaction*													
Overall, I am satisfied with Course One.	100	100	96		Overall, I am satisfied with Course Two.	98	100	98		Overall, I am satisfied with Course Three.	100	100	100
I am satisfied with the format of the course (e.g., group activities, delivery of content).	98	100	96		I am satisfied with the format of the course (e.g., group activities, delivery of content).	93	100	98		I am satisfied with the format of the course (e.g., group activities, delivery of content).	95	100	100
I am satisfied with the content of the course (e.g., topics, currency, relevance).	95	97	98		I am satisfied with the content of the course (e.g., topics, currency, relevance).	95	100	98		I am satisfied with the content of the course (e.g., topics, currency, relevance).	93	97	100
The course materials (readings, participant guide, references) contributed to my learning.	100	97	100		The course materials (readings, participant guide, references) contributed to my learning.	93	100	98		The course materials (readings, participant guide, references) contributed to my learning.	100	100	100
The course was implemented with high quality by faculty and guest speakers.	100	100	100		The course was implemented with high quality by faculty and guest speakers.	98	100	98		The course was implemented with high quality by faculty and guest speakers.	100	100	100
The knowledge and skills I gained are applicable to my professional practice.	100	100	98		The knowledge and skills I gained are applicable to my professional practice.	98	100	98		The knowledge and skills I gained are applicable to my professional practice.	93	100	100
Faculty and staff were responsive to participant questions and concerns.	100	97	100		Faculty and staff were responsive to participant questions and concerns.	95	100	98		Faculty and staff were responsive to participant questions and concerns.	100	100	100
Faculty and guest speakers’ presentation styles were focused, clear, and engaging.	100	97	98		Faculty and guest speakers’ presentation styles were focused, clear, and engaging.	93	97	98		Faculty and guest speakers’ presentation styles were focused, clear, and engaging.	98	100	98
Faculty and guest speakers demonstrated a thorough knowledge base and expertise.	100	100	100		Faculty and guest speakers demonstrated a thorough knowledge base and expertise.	95	100	98		Faculty and guest speakers demonstrated a thorough knowledge base and expertise.	100	100	100
The course met my expectations based on the promotional materials I saw in advance.	95	91	96		The course met my expectations based on the promotional materials I saw in advance.	93	97	96		The course met my expectations based on the promotional materials I saw in advance.	95	91	96
I was satisfied with the registration process for this certificate program.	98	91	98										
*Evaluation target: Kirkpatrick model level 2—learning*													
As a result of Course One, I can name and differentiate between competencies needed to co-create and engage with a range of implementation partners.	98	94	96		As a result of Course Two, I can name and differentiate between competencies needed to support ongoing improvement of an implementation effort.	95	97	98		As a result of Course Three, I can name and differentiate between competencies needed to sustain an implementation or change effort.	100	100	100
As a result of Course One, I can demonstrate an ability to foster co-learning using a one-to-one activity.	93	97	96		As a result of Course Two, I can explain a process for assessing contextual fit and implementation feasibility of a program, practice or strategy and apply it to a case study.	93	97	98		As a result of Course Three, I can compare and contrast four types of psychological safety for teams including strategies used to build each type of psychological safety and the various impacts when they're missing.	93	100	98
As a result of Course One, I can give examples of technical and relational strategies for building trusting relationships.	98	88	96		As a result of Course Two, I can give examples of implementation determinants and implementation strategies matched to those determinants.	86	91	91		As a result of Course Three, I can recognize the characteristics of effective champions and consider these characteristics when identifying champions.	98	100	96
As a result of Course One, I can plan for feasible opportunities to address power differentials and advance equity in my implementation effort.	73	82	94		As a result of Course Two, I can develop a plan that matches implementation strategies to specific implementation challenges.	79	91	93		As a result of Course Three, I can explore a process for assessing and planning for sustainability of a implementation effort using the Sustainability Planning Tool.	88	100	98
As a result of Course One, I can use the spectrum of engagement to identify current levels of engagement for implementation partners and assess whether a different level of engagement is needed.	98	91	94		As a result of Course Two, I can explain the importance of visually communicating data to guide improvement and decision-making.	98	97	87		As a result of Course Three, I can name courageous leadership skills and identify strategies to support use of these skills across implementation teams.	100	100	100
As a result of Course One, I can distinguish how brokering and tailoring support strategies contributed to outcomes in a case example of a change effort.	93	100	91		As a result of Course Two, I can examine key activities used to carry out improvement cycles as illustrated by a case example.	88	94	96		As a result of Course Three, I can describe how values can be used to drive implementation decision-making and examine how values have facilitated or hindered a current implementation effort.	100	97	100
As a result of Course One, I can employ empathy interviews as a method for tailoring support.	88	97	96		As a result of Course Two, I can choose specific facilitation techniques based on intended meeting goals.	88	97	98		As a result of Course Three, I can give examples of strategies used to build implementation capacity and relate them to specific outcomes demonstrating whether capacity has been built.	90	97	96
*Evaluation target: COM-B elements (capability, opportunity, and motivation)*													
I feel capable of using what I’ve learned from Course One (competencies related to Co-Creation and Engagement) in my work supporting implementation.	100	97	98		I feel capable of using what I’ve learned from Course Two (competencies related to Ongoing Improvement) in my work supporting implementation.	93	97	93		I feel capable of using what I’ve learned from Course Three (competencies related to Sustaining Change) in my work supporting implementation.	100	97	96
I believe what I’ve learned from Course One will benefit my work supporting implementation.	100	100	98		I believe what I’ve learned from Course Two will benefit my work supporting implementation.	98	100	100		I believe what I’ve learned from Course Three will benefit my work supporting implementation.	98	100	100
I am motivated to use what I’ve learned from Course One in my work supporting implementation.	100	100	98		I am motivated to use what I’ve learned from Course Two in my work supporting implementation.	98	100	100		I am motivated to use what I’ve learned from Course Three in my work supporting implementation.	98	100	100
I am confident that I will have opportunities to use what I've learned from Course One in my day-to-day work	NA^a^	97	98		I am confident that I will have opportunities to use what I've learned from Course Two in my day-to-day work	98	97	96		I am confident that I will have opportunities to use what I’ve learned from Course Three in my day-to-day work supporting implementation.	95	97	96
I have the supports and resources I need to use what I’ve learned from Course One in my work supporting implementation.	90%	94%	89%		I have the supports and resources I need to use what I’ve learned from Course Two in my work supporting implementation.	88	94	96		I have the supports and resources I need to use what I’ve learned from Course Three in my work supporting implementation.	88	91	93

Note: A/SA = Agree or Strongly Agree.

a This item was not added until courses two and three of cohort 1.

#### Implementation Support Competencies Assessment (ISCA)

Tables 5–7 provide detailed paired-samples t-test results for each cohort. Post-program ISCA completion was encouraged but not required for Cohort 1, resulting in smaller sample sizes (n = 10–11); it became a formal requirement for Cohorts 2 and 3, yielding higher response rates (n = 28 and n = 41, respectively). Results indicated statistically significant increases from pre- to post-program across all 15 core competencies in all three cohorts (*p* < .05 for Cohort 1; *p* < .001 for Cohorts 2 and 3), with large effect sizes (standardized mean differences ranging from 0.80 to 2.40).

**Table 5.** Comparison of Pre- and Post-program Implementation Support Competencies Assessment (ISCA) Scores for Cohort 1.

		Number of items		Internal consistency reliability^a^			Pre-program score^b^	Post-program score^b^	Mean difference	SMD			
#	Competency			α	Ω	*n*					% change	t-statistic	*p*-value
*Domain: co-creation and engagement*													
1	Co-learning	6		0.70	0.72	11	3.35	3.91	0.56	1.59	17	5.29	<.001
2	Brokering	6		0.80	0.80	11	3.26	3.94	0.68	1.12	21	3.72	.004
3	Address power differentials	7		0.87	0.87	11	3.19	4.10	0.91	2.40	28	7.95	<.001
4	Co-design	6		0.89	0.89	11	2.83	3.85	1.02	1.35	36	4.48	.001
5	Tailoring support	7		0.93	0.93	11	2.96	3.96	1.00	1.21	34	4.02	.002
*Domain: ongoing improvement*													
6	Assess needs and assets	6		0.74	0.70	11	3.20	4.05	0.85	1.77	27	5.86	<.001
7	Understand context	6		0.85	0.84	11	3.09	3.86	0.77	1.92	25	4.80	<.001
8	Apply and integrate implementation frameworks, strategies, and approaches	5		0.89	0.89	11	3.05	3.73	0.67	0.95	22	3.16	.010
9	Facilitation	9		0.87	0.87	10	3.49	4.18	0.69	1.14	20	3.61	.006
10	Communication	6		0.92	0.93	10	3.13	3.93	0.80	1.01	26	3.19	.010
11	Conduct improvement cycles	6		0.92	0.92	10	3.03	3.93	0.90	1.06	30	3.36	.008
*Domain: sustaining change*													
12	Grow and sustain relationships	11		0.86	0.87	10	3.21	4.05	0.85	1.19	26	3.75	.005
13	Develop teams	15		0.94	0.95	10	3.19	3.89	0.70	0.80	22	2.61	.028
14	Build capacity	8		0.92	0.93	10	3.08	3.91	0.84	1.11	27	3.51	.007
15	Cultivate leaders and champions	9		0.94	0.94	10	3.11	3.94	0.83	0.99	27	3.14	.012

*Note:* α = Cronbach's alpha; Ω = McDonald's omega; ^a^assessments of internal consistency reliability were conducted using pre-program ISCA responses, which included 39 respondents for Co-Creation and Engagement domain, 37 respondents for the Ongoing Improvement domain, and 37 respondents for the Sustaining Change domain. *n* = the number of cases eligible for inclusion in the paired-samples t-tests given available data at both pre- and post-program. ^b^Scores ranged from 1 to 5. SMD = standardized mean difference (i.e., difference between pre- and post-program scores in standard deviation units).

**Table 6.** Comparison of Pre- and Post-program Implementation Support Competencies Assessment (ISCA) Scores for Cohort 2.

		Number of items		Internal consistency reliability^a^			Pre-Program score^b^	Post-program score^b^	Mean difference	SMD			
#	Competency			α	Ω	*n*					% change	t-statistic	*p*-value
*Domain: co-creation and engagement*													
1	Co-learning	6		0.68	0.69	28	2.97	3.99	1.02	1.79	34	9.48	<.001
2	Brokering	6		0.88	0.88	28	3.01	4.07	1.07	1.48	35	7.82	<.001
3	Address power differentials	7		0.84	0.85	28	3.12	4.11	0.99	1.89	32	9.99	<.001
4	Co-design	6		0.86	0.87	28	2.79	4.04	1.24	1.91	45	10.10	<.001
5	Tailoring support	7		0.95	0.95	28	2.94	4.06	1.12	1.50	38	7.94	<.001
*Domain: ongoing improvement*													
6	Assess needs and assets	6		0.80	0.76	28	3.41	4.09	0.68	1.40	20	7.42	<.001
7	Understand context	6		0.87	0.88	28	2.94	3.90	0.96	1.74	33	9.20	<.001
8	Apply and integrate implementation frameworks, strategies, and approaches	5		0.94	0.94	28	2.65	3.59	0.94	1.39	35	7.38	<.001
9	Facilitation	9		0.94	0.94	28	3.42	4.12	0.70	1.00	20	5.27	<.001
10	Communication	6		0.95	0.96	28	3.17	4.01	0.84	1.13	26	5.98	<.001
11	Conduct improvement cycles	6		0.93	0.93	28	3.03	3.88	0.85	1.46	28	7.75	<.001
*Domain: sustaining change*													
12	Grow and sustain relationships	11		0.91	0.92	28	3.65	4.35	0.70	1.32	19	6.96	<.001
13	Develop teams	15		0.95	0.95	28	3.30	4.03	0.72	1.17	22	6.19	<.001
14	Build capacity	8		0.90	0.90	28	3.18	3.89	0.71	1.23	22	6.49	<.001
15	Cultivate leaders and champions	9		0.96	0.96	28	3.33	3.96	0.64	1.02	19	5.41	<.001

*Note:* α = Cronbach's alpha; Ω = McDonald's omega; ^a^assessments of internal consistency reliability were conducted using pre-program ISCA responses, which included 36 respondents for Co-Creation and Engagement domain, 35 respondents for the Ongoing Improvement domain, and 34 respondents for the Sustaining Change domain. *n* = the number of cases eligible for inclusion in the paired-samples t-tests given available data at both pre- and post-program. ^b^Scores ranged from 1 to 5. SMD = standardized mean difference (i.e., difference between pre- and post-program scores in standard deviation units).

**Table 7.** Comparison of Pre- and Post-program Implementation Support Competencies Assessment (ISCA) Scores for Cohort 3.

		Number of items		Internal consistency reliability^a^			Pre-program score^b^	Post-program score^b^	Mean difference	SMD			
#	Competency			α	Ω	*n*					% change	t-statistic	*p*-value
*Domain: co-creation and engagement*													
1	Co-learning	6		0.85	0.85	41	3.12	4.04	0.92	1.35	29	8.66	<.001
2	Brokering	6		0.90	0.90	41	2.98	4.14	1.16	1.44	39	9.19	<.001
3	Address power differentials	7		0.93	0.93	41	3.02	4.09	1.08	1.59	36	10.21	<.001
4	Co-design	6		0.93	0.93	41	2.95	4.07	1.12	1.31	38	8.39	<.001
5	Tailoring support	7		0.94	0.94	41	2.84	4.07	1.23	1.48	43	9.48	<.001
*Domain: ongoing improvement*													
6	Assess needs and assets	6		0.87	0.88	41	2.91	4.05	1.14	1.32	39	8.46	<.001
7	Understand context	6		0.92	0.92	41	2.74	4.01	1.27	1.49	47	9.54	<.001
8	Apply and integrate implementation frameworks, strategies, and approaches	5		0.93	0.93	41	2.51	3.92	1.41	1.62	56	10.38	<.001
9	Facilitation	9		0.96	0.96	41	3.12	4.19	1.07	1.33	34	8.49	<.001
10	Communication	6		0.95	0.95	41	2.76	4.04	1.28	1.31	47	8.37	<.001
11	Conduct improvement cycles	6		0.95	0.95	41	2.68	3.98	1.30	1.57	48	10.05	<.001
*Domain: sustaining change*													
12	Grow and sustain relationships	11		0.96	0.96	41	3.31	4.23	0.92	1.42	28	9.12	<.001
13	Develop teams	15		0.96	0.96	41	2.85	4.04	1.19	1.67	42	10.70	<.001
14	Build capacity	8		0.95	0.95	41	2.83	3.98	1.15	1.53	41	9.82	<.001
15	Cultivate leaders and champions	9		0.96	0.96	41	2.86	3.95	1.08	1.34	38	8.57	<.001

*Note:* α = Cronbach's alpha; Ω = McDonald's omega; ^a^assessments of internal consistency reliability were conducted using pre-program ISCA responses, which included 47 respondents for all three domains. *n* = the number of cases eligible for inclusion in the paired-samples t-tests given available data at both pre- and post-program. ^b^Scores ranged from 1 to 5. SMD = standardized mean difference (i.e., difference between pre- and post-program scores in standard deviation units).

Examining improvement patterns by domain, co-creation and engagement competencies (Course 1; five competencies) increased 17%–45% across cohorts. The largest gains occurred for co-design (36%, 45%, and 38% across Cohorts 1, 2, and 3, respectively), whereas the smallest gains occurred for co-learning (17%, 34%, and 29%). Ongoing improvement competencies (Course 2; six competencies) increased 20%–56% across cohorts. Notably, the “apply and integrate implementation frameworks, strategies, and approaches” competency showed substantial gains across all cohorts (22%, 35%, and 56%), with Cohort 3 demonstrating the largest improvement. Facilitation showed the most consistent gains (20%, 20%, and 34%). Sustaining change competencies (Course 3; four competencies) increased 19–42% across cohorts. The “develop teams” competency showed the largest range of gains (22%, 22%, and 42%), whereas grow and sustain relationships showed more consistent gains (26%, 19%, and 28%). Overall, the sustaining change domain demonstrated more consistent improvement patterns across cohorts compared to the other two domains.

#### Follow-Up Surveys

Participants from Cohort 1 (n = 23; 1 year post-completion) and Cohort 2 (n = 23; three months post-completion) rated the perceived impact of all three courses highly. For Cohort 1, mean scores were 4.00 (standard deviation [SD] = 1.09) for Course 1, 3.96 (SD = 0.98) for Course 2, and 3.96 (SD = 1.02) for Course 3. For Cohort 2, mean scores were 3.96 (SD = 0.82) for Course 1, 4.09 (SD = 0.85) for Course 2, and 4.17 (SD = 0.83) for Course 3, where response options ranged from 1 (*not at all impactful*) to 5 (*extremely impactful*). Impact ratings were comparable between cohorts despite different follow-up intervals, suggesting sustained program value over time.

### Qualitative Findings

#### What Participants Liked Most and Suggestions for Program Improvement

Our analysis of participants’ responses related to what they liked most about the program and courses yielded several themes. One theme centered on participants’ appraisal of the certificate program faculty, guest speakers, and content as being high quality. On this front, participants noted that in addition to possessing substantive expertise, program faculty and guest speakers modeled key facilitation skills and conveyed respect and empathy for each other and for program participants. Another theme captured positive reactions to the overall format of the certificate program. Participants reported benefiting from the synchronous approach to online content delivery; as well as the mixture of presentations, small group reflection and application activities, and large group discussion. Small-group engagements particularly were highlighted as a beneficial component of the certificate program. Participants emphasized the positive impact of small-group work and enjoyed having consistent small-group membership for the duration of the certificate program. Another theme reflected how an explicit focus on equity throughout the certificate program added value to participants’ learning and experience. Many participants indicated feeling energized to take action and transform systems as a result of course content and activities that incorporated an equity focus. These dynamics were fortified by activities that enabled participants to articulate and share with each other the values that motivated them to engage in their current implementation work. Another theme centered on participants’ assessment that program content was highly relevant and directly applicable to their work. As one participant stated succinctly: “Very practical. I can use all of it tomorrow.”

In terms of program improvement, participants expressed the potential value in expanding the amount of time spent in small groups. Feedback of this sort was shared following all three program courses. Participants also noted that it would be helpful for speakers—including guest speakers—to explicitly highlight the course-preparation materials (e.g., articles) that were connected with the content being shared.

#### Sustained Value and Ongoing Use of Program Content

Open-ended responses from the follow-up survey (cohorts 1 and 2) highlighted participants’ ongoing use of program content in the context of their work. For example, participants reported successful application of course content and tools related to assessing the contextual fit and feasibility of specific evidence-informed interventions, developing implementation teams, and fostering psychological safety among implementation partners. Weeks or months after program completion, some participants noted retaining feelings of validation, courage, self-confidence, and gratitude. Participants also noted that they retained a sense of community and belonging following program completion in what was described to be the “lonely practice of implementation support.” Participants reported numerous additional impacts over time linked to program completion, including the following: common language for internal teams to utilize together and a supportive team dynamic to digest and integrate learnings in daily practice (specific to closed cohort); new and effective ways of approaching implementation support work; refinement of interventions to promote successful implementation; integration of resources, tools, and learnings into existing work plans; increased competence to effectively engage in implementation support work; increased trust and psychological safety on internal and external teams; increased intentionality and reflection in daily practice; development of materials to share learnings with team members and other professionals; transfer of knowledge to team members and other professionals; greater organization and structure regarding implementation practice as a profession; buy-in and a sense of mutual ownership on implementation teams; greater awareness of inequity and confidence addressing it; and increased confidence advocating for relational infrastructure to support implementation support work.

## Discussion

Core competencies were used to inform the development of the university-based certificate program in implementation practice, with an emphasis on experiential learning and skill-building related to each competency. Recruitment efforts for the program also focused specifically on implementation support practitioners with training program content and activities tailored to professionals who serve in this implementation support role. Participants in the certificate program reported (a) notable levels of satisfaction with course content and learning experiences; (b) the acquisition of relevant knowledge; (c) high levels of perceived capability, motivation, and opportunity to apply learnings; and (d) significant and sizable gains across all 15 measured competencies. Efforts to follow-up with program participants produced evidence of course content application and notable sustained impacts on participants’ implementation support work, with positive spillover into participants’ teams and work organizations.

Participants’ reflections about how the program shifted their perceptions of self and professional role in the context of implementation suggest that a certificate program tailored to the specific role of implementation support practitioners might help participants expedite their pathway to effective implementation support practice. Metz and colleagues (Metz, Jensen, Farley, and Boaz, 2022) described in a previous study that implementation support practitioners use a broad set of competencies, undergirded by relational and technical skills, to effectively bridge the research-to-practice gap through co-creation—the ultimate goal of implementation science. As such, this certificate program embraced the breadth of this role and provided capacity-building on technical skills, such as using data for continuous improvement of implementation strategies, and relational skills, such as building teams, cultivating champions, and growing and sustaining trusting relationships. Certificate program participants reflected on the resonance of this skill-building for their role supporting implementation and their increased commitment and motivation to use what they learned to improve their implementation support activities in this role.

Certificate program content emphasized the intersectionality of relational and technical strategies, describing, for example, how building trust can ensure that data use activities are more productive and inclusive. Study findings signal that certificate program participants acquired an increased appreciation for the role of relational dynamics in supporting implementation, which affirmed their personal experiences developing trust between themselves and implementation partners and fostering trust among implementation partners (Metz, Jensen, et al., 2024; Metz, Jensen, Farley, & Boaz, 2022; Metz, Jensen, Farley, Boaz, Bartley, et al., 2022).

Qualitative insights also foreground the role of relational and interpersonal factors modeled by program faculty in shaping a high-quality learning environment. These findings speak to an underexplored area of study related to the role of program faculty in modeling specific values and competencies for implementation support in their interactions with each other and with program participants. In particular, program participants experienced empathy from and among program faculty. As issues of resilience among implementation support practitioners garner more attention (Olmos-Ochoa et al., 2021), it is possible that training and capacity-building programs that provide empathy and support for program participants have the added benefit of fostering resilience among members of the implementation support workforce by instilling hope and creating a nurturing environment. Indeed, program participants emphasized the learning community and relationships that were created through the certificate program and expressed an interest in continuing to be a part of this community.

The long waitlists for this certificate program during each enrollment period signal an ongoing need in the field of implementation science to increase the availability of and access to training for professionals who seek to increase their knowledge and skills in implementation practice specifically. Other training programs currently available help to meet this need and there is ample room for all training programs to effectively contribute to the development of an implementation practice workforce. Indeed, the development of more training programs would be beneficial. Although the purpose of this study was not to formally compare this certificate program with other training programs focused on implementation practice, the program featured here potentially offers a valuable contribution to the ecosystem of implementation science training and certificate opportunities. The program is built on an extensive and recent research base for competencies for implementation support practitioners; it's the first program to assess whether training can produce changes in scores from the psychometrically validated ISCA (Jensen et al., 2024); it focuses on values and competencies, rather than specific methods (e.g., Plan-Do-Study-Act Cycles or evaluation methods); it supports the integrative use of implementation science theories, models, and frameworks broadly with a set of practice skills to bring these theories, models, and frameworks to life in day-to-day service settings; and it is not focused on a specific service sector (e.g., healthcare) and seeks to build a professional workforce across a range of health and human service and early childhood and education contexts.

Program participants shared that there might be value in generating asynchronous pre-course modules that cover some basic topics in preparation for the program or having participants directed to such content that already exists. Asynchronous learning could serve as a condition for scaling the implementation practice certificate program which has generated long waitlists (hundreds of individuals). Moving forward, it could be informative to assess the efficacy of asynchronous content delivery in relation to the notable value brought to participants via the synchronous sessions, small breakout groups, and emphasis on skill acquisition and relationship development.

Although the current study offers initial evidentiary support of participants’ satisfaction, learning, and competency acquisition and application, limitations should appropriately temper conclusions. For one, sample sizes across cohorts were small and our research design (e.g., no treatment randomization) was unable to account for potential selection effects (e.g., the characteristics of those who enrolled in the program could be partial drivers of observed outcomes). In addition, although we did include measures in follow-up surveys to assess the sustained value and ongoing use of program content by participants in their day-to-day work, our study did not include data pertaining to long-term implementation outcomes associated with participants’ acquisition and application of implementation knowledge and skills. Future research could endeavor to bolster the research design (e.g., waitlist control design) to derive more valid counterfactual conditions for evaluating the efficacy of the certificate program, while also incorporating longer-term follow-up measures to assess downstream, real-world implementation outcomes.

## Conclusions

Taken together, our evaluation of a university-based, competency-focused certificate program intended to bolster the workforce of implementation support practitioners yielded promising results. Indeed, a large majority of program participants indicated their satisfaction with course content and reported notable and sustained gains in implementation-related knowledge, skills, motivation, capability, and opportunity. Building the implementation workforce can happen in different ways—through the development of professionals who reside outside the systems they support (e.g., intermediaries, university-based implementation centers, independent professionals), and through the growth of professionals who already have fully developed careers within the systems they manage. This certificate program embraces both of these pathways, thereby increasing the likelihood of growing the workforce and reducing the risk associated with a single pathway for developing the profession of implementation support.

This study also highlighted the efficacy and value of online, synchronous delivery of course content, with small-group engagements being an especially beneficial learning environment. It also is notable that the certificate program enabled meaningful shifts in how participants viewed themselves and their role in implementation settings, with a deepened appreciation for the power of co-learning and co-creation processes; as well as the relational, interpersonal, and intrapersonal elements that can optimize sustainable implementation efforts in combination with well-established implementation theories, models, frameworks, and technical strategies.
